# Neutrophil extracellular traps are indirectly triggered by lipopolysaccharide and contribute to acute lung injury

**DOI:** 10.1038/srep37252

**Published:** 2016-11-16

**Authors:** Shuai Liu, Xiaoli Su, Pinhua Pan, Lemeng Zhang, Yongbin Hu, Hongyi Tan, Dongdong Wu, Ben Liu, Haitao Li, Haosi Li, Yi Li, Minhui Dai, Yuanyuan Li, Chengping Hu, Allan Tsung

**Affiliations:** 1 Department of Pulmonary and Critical Care Medicine, Xiangya Hospital, Central South University, Changsha, Hunan 410008, China; 2 Department of Thoracic Medicine, Hunan Cancer Hospital, Affiliated to Xiangya Medical School, Central South University, Changsha, Hunan 410013, China; 3 Department of Pathology, Xiangya Hospital, Central South University, Changsha 410008, China; 4 Department of Surgery, University of Pittsburgh, Pittsburgh PA, 15213, USA

## Abstract

Neutrophil extracellular traps (NETs) facilitate the extracellular killing of pathogens. However, excessive NETs formation and poor degradation are associated with exacerbated immune responses and tissue injury. In this study, we investigated the role of NETs in lipopolysaccharide (LPS)-mediated acute lung injury (ALI) and assessed the use of DNase I, for the treatment of ALI. Additionally, we focused on the controversial issue of whether LPS directly induces NETs release *in vitro*. NETs formation was detected in murine ALI tissue *in vivo* and was associated with increased NETs markers, citrullinated-histone H3 tissue levels and NET-DNA levels in BALF. Treatment with DNase I significantly degraded NETs and reduced citrullinated-histone H3 levels, which protected against ALI and ameliorated pulmonary oedema and total protein in BALF. In addition, DNase I significantly reduced IL-6 and TNF-α levels in plasma and BALF. *In vitro*, LPS-activated platelets rather than LPS alone efficiently induced NETs release. In conclusion, NETs formed during LPS-induced ALI, caused organ damage and initiated the inflammatory response. NETs degradation by DNase I promoted NET-protein clearance and protected against ALI in mice; thus, DNase I may be a new potential adjuvant for ALI therapy. Specifically, LPS induced NETs formation in an indirect manner via platelets activation.

Acute lung injury and acute respiratory distress syndrome (ALI/ARDS) are complex and severe lung disorders associated with a high mortality rate in critically ill patients^1^,^2^. Despite the use of mechanical ventilation with a low tidal volume, it is difficult to effectively prevent, reduce and treat ALI/ARDS^3^. Neutrophils are among the most important innate immune cells in human beings. Neutrophil activation, infiltration and delayed clearance are thought to play essential roles in the pathogenesis of ALI/ARDS. Neutrophil recruitment into the lung parenchyma and alveolar space, induced by chemokines secreted by macrophages, epithelial cells, and neutrophils themselves, has been observed during infections associated with ALI/ARDS. Phagocytosis and degranulation are common mechanisms employed by neutrophils to kill pathogens. Additionally, a novel antibacterial strategy that localizes to and eliminates pathogens, called neutrophil extracellular traps (NETs), has been described^4^.

NETs are net-like chromatin fibres decorated with neutrophil-derived antimicrobial proteins generated by dying neutrophils through a distinct process termed NETosis. NETs immobilize or trap various pathogens, subsequently counteracting the dissemination of infections and ultimately promoting pathogen killing^4^,^5^. However, the excessive presence of NETs and, particularly, NET-bound components also results in harmful effects. For example, histones and myeloperoxidase (MPO) are cytotoxic to epithelial and endothelial cells^6^. Similarly, neutrophil elastase (NE) increases the permeability of the alveolar-capillary barrier by cleaving the endothelial actin cytoskeleton, VE-cadherin and E-cadherin. Proteinase 3 (PR3) and cathepsin G (Cat G) enhance inflammatory reactions by activating proinflammatory proteins and degrading anti-inflammatory proteins^7^. LL-37, an antibacterial protein detected in NETs structures, increases activation of the NLRP3 inflammasome in adjacent macrophages, resulting in the release of inflammatory cytokines^8^. Reactive oxygen species (ROS) produced by MPO create an injurious environment for epithelial cells, which causes their apoptosis or necrosis^7^. Furthermore, NETs are potent drivers of the coagulation cascade and disrupt the microcirculation, which mediate organ failure during sepsis^9^. In addition, NETs are involved not only in subsequent fibrosis but also in angiogenesis^10^,^11^, which may contribute to tissue remodelling during the later phases of ALI/ARDS. Overall, accumulating evidence supports the biological relevance of NETs in the pathogenesis of ALI/ARDS.

Lipopolysaccharides (LPS) are essential constituents of the outer membranes of Gram-negative bacteria, which have been widely applied to establish ALI models. It is unclear whether LPS causes NETs release, although LPS induces NETs formation had been reported^4^. Conversely, according to Clark^9^and his colleagues, LPS does not cause NETs release upon direct contact with neutrophils; otherwise, LPS-activated platelets would effectively induce NETs production. Furthermore, recent studies investigating NETs have been primarily confined to *in vitro* experiments, and the *in vivo* role of NETs in ALI/ARDS remains unclear. In this article, we explored whether LPS is able to induce NETs release both *in vivo* and *in vitro*, identified the role of NETs in ALI by applying deoxyribonuclease I (DNase I) and formulated a new therapeutic strategy for ALI/ARDS.

## Results

### NETs Formation in LPS-induced ALI

**Figure 1:**
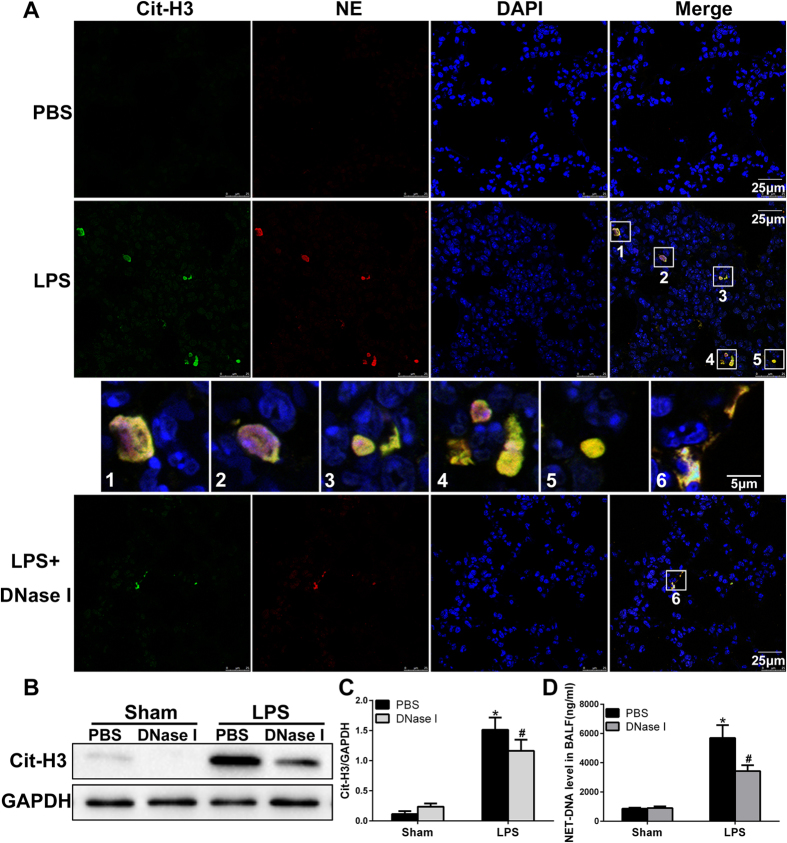
NETs are formed in an LPS-induced ALI mouse model. (**A**) Representative immunofluorescence images obtained by performing confocal microscopy of lung sections from mice(n = 6) 24 h after intratracheal LPS treatment, compared to sham and DNase I plus LPS groups; staining depicts Cit-H3 (green), NE(red) and DAPI (blue). The higher-magnification views in the insets (1–6) show NETs formation, demonstrated by the co-localization of Cit-H3 (green), NE (red) and DNA (blue). (**B**,**C**) Western blot analysis of Cit-H3 protein levels in the lungs of sham-, LPS-, or DNase I plus LPS-treated mice. Full-length blots/gels are presented in Supplementary Figure S1. (**D**) BALF levels of NET-DNA were determined using a PicoGreen assay kit. *P < 0.05 vs. the sham group; ^#^P < 0.05 vs. the LPS group. The results shown are representative of at least three separate independent experiments.

To investigate whether migratory neutrophils form NETs in LPS-induced ALI, we first performed immunofluorescence staining of paraffin-embedded mouse lung sections. NETs formation was detected in mouse lung tissue after LPS administration but was not observed in phosphate-buffered saline (PBS) control mice. Notably, small amounts of NETs were present in tissues from LPS-exposed animals pretreated with DNase I (Fig. 1A). We identified NETs based on the co-localization of extracellular fibrillary and web-like structures with citrullinated-histone H3 (Cit-H3) as well as the appearance of neutrophil-derived NE. Importantly, NETs detection was strongly associated with DNA/histone antibodies rather than decondensed chromatin, which was weakly stained by DAPI. However, NETs detected in the lung tissue lacked mesh-like extracellular chromatin structures, perhaps due to the space limitations of the lung parenchyma and the presence of short fragments of DNA-protein complexes in the airways^12^. In the next set of experiments, we analysed Cit-H3(another specific marker of NETs formation) levels by performing western blotting and observed significantly higher levels of Cit-H3 in the lung tissues from LPS-treated mice than those in the lung tissues from mice in the sham group. In addition, lung tissue from DNase I-treated mice contained significantly lower levels of Cit-H3 than mice exposed to LPS; however, the administration of DNase I did not completely eliminate Cit-H3 (Fig. 1B,C). To further confirm NETs formation within the lungs, we also measured NET-DNA levels in BALF; NET-DNA is an additional marker of NETs formation. NET-DNA levels in the BALF of mice undergoing ALI were significantly higher than those in the sham group. Treatment with DNase I significantly decreased the amount of NET-DNA in the BALF of mice administered LPS (Fig. 1D).

### NETs Degradation by DNase I Attenuates LPS-mediated ALI in Mice

**Figure 2:**
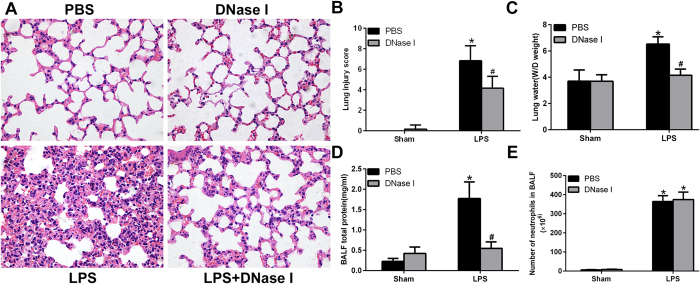
NETs regulate tissue damage during ALI. Mice were treated with 20 μl of 3 mg/ml DNase I at time 0 and 10 h after LPS instillation (4 mg/kg in 25 μl of PBS), and lung samples and BALF were collected from the mice 24 h after LPS treatment. (**A**) H&E staining of lung sections (400×); (**B**) Lung injury scores; (**C**) lung water content; (**D**) total protein concentration in BALF. (**E**) The number of alveolar neutrophils in BALF; *P < 0.05 vs. the sham group; ^#^P < 0.05 vs. the LPS group. n = 6 mice/group. The results are representative of three separate independent experiments.

We next evaluated whether NETs formation was pathogenic in LPS-mediated ALI. To degrade NETs, we treated mice with DNase I, which effectively catalyses hydrolysis of the NETs backbone structure. Histopathological analysis of lung tissues revealed severe destruction of the lung structures following LPS administration for 24 h, typified by the infiltration of inflammatory cells, haemorrhage and interstitial oedema. Exogenously administered DNaseI significantly reduced haemorrhage and oedema (Fig. 2A). Lung injury scores (Fig. 2B), pulmonary wet/dry (W/D) weights (Fig. 2C) and total proteins in BALF (Fig. 2D) were significantly higher in the LPS group than in the sham group. DNase I treatment significantly reduced LPS-provoked lung injury, measured by the lung injury score, pulmonary W/D weight and total proteins in BALF, which was consistent with decreased NET-DNA levels in BALF and Cit-H3 levels in lung tissues. Notably, however, the administration of DNase I did not alter neutrophil infiltration into BALF (Fig. 2E), suggesting neutrophil recruitment to the site of injury is barely disturbed although NETs degradation reduces organ injury.

### NETs Regulate Systemic and Local Inflammation

**Figure 3:**
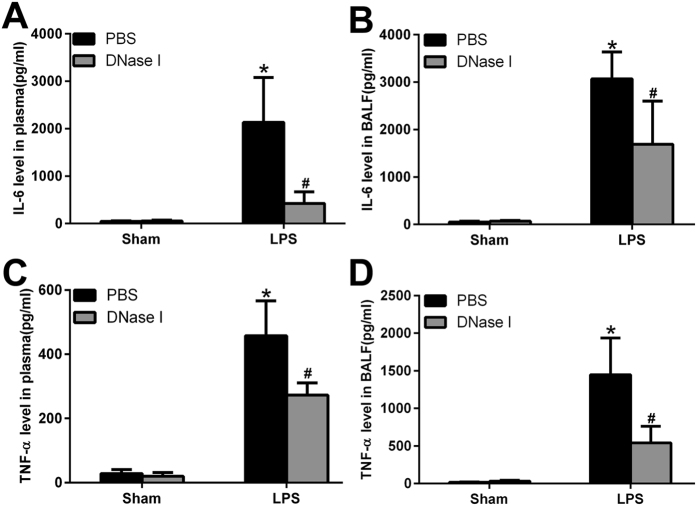
NETs regulate inflammatory responses both in BALF and plasma. After DNase I treatment and LPS exposure at predetermined time points, IL 6 and TNF-α levels in the plasma (**A,C**) and BALF (**B,D**) were measured. *P < 0.05 vs. the sham group; ^#^P < 0.05 vs. the LPS group. n = 6 mice/group. All data shown are representative of at least three separate independent experiments.

Excessive NETs formation and poor clearance may induce severe inflammatory processes involving leukocyte accumulation, diffuse alveolar damage and epithelial injury, which result in the massive release of inflammatory cytokines both locally and in the circulation via increased alveolar permeability. To test this hypothesis, we measured cytokine levels (IL-6, TNF-α) in plasma and BALF. IL-6 and TNF-α levels in plasma and BALF were low but detectable in sham animals, while the administration of DNase I alone had no effects on IL-6 and TNF-α levels. In contrast, IL-6 and TNF-α levels in plasma and BALF were markedly elevated in LPS-challenged mice, and treatment with DNase I reduced IL-6 and TNF-α levels in these animals (Fig. 3).

### LPS Indirectly Promotes the NETs Release

**Figure 4:**
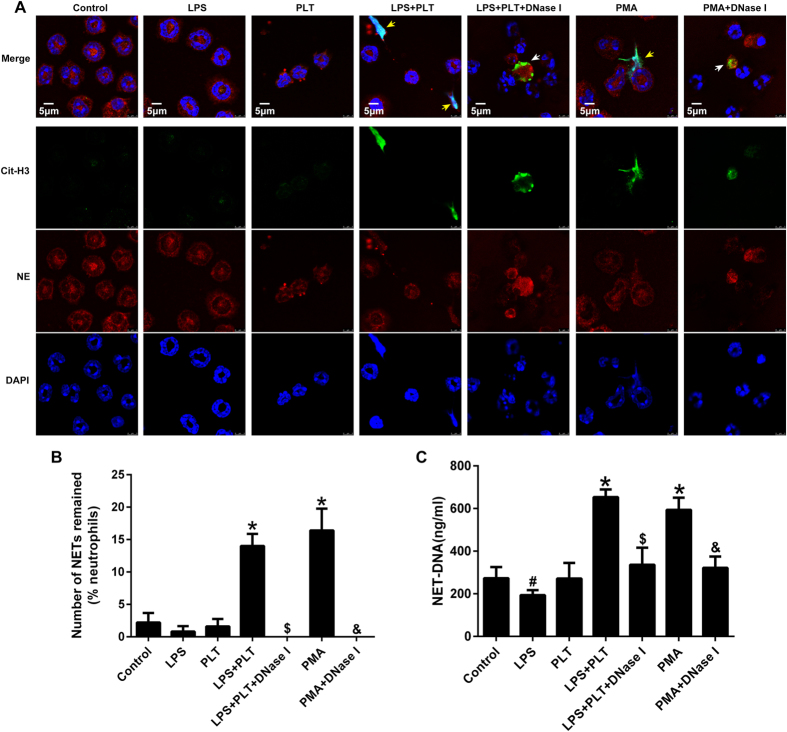
Activated platelets, rather than LPS alone, induce NETs release. Neutrophils (10^5^) from the bone marrow were left untreated or stimulated with 100 nM PMA as negative and positive controls for NETs formation, respectively. In addition, neutrophils were co-cultured with LPS (5 μg/ml), PLT (10^6^), LPS plus PLT, LPS plus PLT plus DNase I(200 U/ml), or PMA plus DNase I for 4 h. (**A**) Immunofluorescent staining of NETs detected by confocal microscopy in neutrophils. Yellow arrows show NETs formation, white arrows show the remnants of NETs processed by DNase I. Green: Cit-H3; red: NE; blue: nuclei. (**B**) Fluorescence microscopy images were analysed with Image J software to count the number of NETs remained per one hundred neutrophils; a zero indicates no intact NETs were observed. (**C**) NET-DNA levels in cell supernatants following neutrophil exposure to various stimuli were quantified using the PicoGreen assay kit. *P < 0.05 vs. the control, LPS or PLT groups; ^#^P < 0.05 vs. the control group; ^$^P < 0.05 vs. the LPS plus PLT group; ^&^P < 0.05 vs. the PMA group. All data shown are representative of at least three separate independent experiments.

It is controversial whether LPS is able to induce NETs release. In our experiments, using phorbol 12-myristate 13-acetate (PMA) as a positive control, LPS alone was unable to induce NETs release during direct contact with neutrophils; conversely, LPS-activated platelets efficiently elicited NETs generation. Of note, DNase I treatment destroyed the NETs structure, while having no effect on Cit-H3 and NE (Fig. 4A,B). Interestingly, NET-DNA levels decreased when neutrophils came into contact with LPS alone, which may be related to the ability of LPS to inhibit neutrophil apoptosis and prolong the life span of these cells (Fig. 4C). Activated platelets that interact with neutrophils are partially dependent on Toll-like receptor 4 (TLR4) and present the high mobility group box 1 (HMGB1) to neutrophils, resulting in NETs generation^9^,^13^. Taken together, LPS is sufficient to commit neutrophils to NETs production in our model of ALI; however, this process does not occur through the direct interaction of LPS and neutrophils but instead is due to activated platelets or the inflammatory environment created by LPS, which may be more important for NETosis.

## Discussion

ALI/ARDS encompasses acute diffuse inflammation of the lungs caused by a multitude of direct and indirect pathogenic factors; this inflammation is characterized by the impairment of alveolar-capillary barrier function, the exudation of protein-rich fluid into the alveoli, oedema and formation of the hyaline membrane, leading to acute respiratory failure^14^. In this study, we observed an increase in NETs formation during LPS-induced ALI in mice, demonstrated by the wide-spread co-localization of DNA, NE and Cit-H3 revealed by immunofluorescence staining. Moreover, LPS challenge increased Cit-H3 levels in lung tissues and NET-DNA in BALF. Theoretically, NET-associated proteins should remain when DNA structures are disrupted by DNase I, which specifically degrades NET-DNA but not NET-derived proteins. Interestingly, the transtracheal instillation of DNase I resulted in decreased levels of Cit-H3 and BALF NET-DNA but only partially, rather than completely, reduced NETs generation. This may occur because DNase I splits only NETs formed by neutrophil exudation into the alveoli rather than capillary-associated NETs. Alternatively, NETs processing by DNase I may facilitate their clearance by macrophages^15^,^16^.

NETs bind to and promote the killing of bacteria, fungi and Leishmania parasites when they are released into local tissue or the circulation. However, excessive NETs generation results in damaging effects and impaired tissue function^6^,^9^,^17^. As reported in this study, NETs digestion by DNase I significantly ameliorated tissue injury and the degree of systemic and local inflammation, providing evidence to support the contribution of NETs to lung injury. NET-derived histones are highly toxic to epithelial and endothelial cells^6^, and the decrease in histone levels following NETs degradation by DNase I may explain why this drug mitigates lung tissue injury^18^. LPS increased IL-6 and TNF-α levels in the circulation and BALF, possibly because NETs in the alveoli interacted with resident tissue cells and immune cells that infiltrated into the lungs, resulting in increased pro-inflammatory cytokine levels in BALF as well as their release into the circulation. Although DNase I disturbed NETs structures and promoted NETs clearance by macrophages, it did not induce proinflammatory cytokine secretion^16^, potentially resulting in decreased levels of IL-6 and TNF-α; this suggests NETs possess the ability to regulate systemic and local inflammation during ALI. Treatment with recombinant human DNase improves airflow obstruction and decreases the rate of infectious respiratory exacerbation in some cystic fibrosis patients^19^. In summary, targeting NETs may represent a potentially effective method to treat ALI.

NETosis is triggered by neutrophil exposure to PMA, interferon-gamma(IFN-γ), Leishmania, bacteria, viruses and fungi and their products, and complement-mediated opsonization^4^,^20^,^21^,^22^,^23^. The percentage of activated neutrophils that undergo NETosis, the rapidity of NETs release, and the mediators and molecular pathways involved in NETosis vary based on the type of stimulus^24^,^25^. Specifically, LPS stimulates NETs generation^4^. However, we did not observe NETs formation when neutrophils were co-cultured with LPS or platelets alone *in vitro*. In addition, the quantity of NET-DNA induced by LPS in cell culture supernatants was decreased compared to the control group, potentially because LPS protects neutrophils against apoptosis and inhibits NETs formation^26^,^27^. LPS induced an increase in NETs formation *in vivo*, although the increased levels of pro-inflammatory molecules (such as IL-6, TNF-α, HMGB1, etc.), platelet activation and other indirect effects of LPS might account for this phenomenon.

Evidence from the literature indicates that both neutrophils and platelets participate in and synergistically contribute to the inflammatory response during sepsis; platelets are recruited to the pulmonary capillaries and liver sinusoids in a neutrophil-dependent manner, leading to reductions in the number of circulating platelets as well as lung and liver dysfunction and/or failure^9^. In our study, neutrophils failed to form NETs when they directly contacted LPS *in vitro*, suggesting that platelets are necessary for NETosis triggered by LPS. TLR4, a member of the TLR family expressed in different cell types, acts as the specific transmembrane receptor of LPS. The interaction of TLR4 with LPS activates a signalling cascade, resulting in the production of pro-inflammatory cytokines and subsequent immune responses that create an inflammatory environment benefitting NETosis. TLR4- and LPS-mediated binding of activated platelets to neutrophils promotes efficient NETs release, in part because activated platelets present HMGB1 to neutrophils and commit them to autophagy and NETs generation^13^.

In conclusion, NETs formed in the trachea during LPS-induced ALI, caused organ damage and initiated an inflammatory response, and the degradation of NETs formation by DNase I promoted NET-protein clearance and protected against ALI in mice. In contrast to our expected results, NETs were induced by LPS-stimulated platelets, suggesting that direct interactions between neutrophils and Gram-negative bacteria may be not necessary for NETs generation. A better understanding of the mechanisms underlying NETs release induced by LPS and the effects of NETs on host immune system modulation will support the development of potential new therapeutic strategies for ALI.

## Materials and Methods

### Animals

Male C57BL/6 mice, aged 8–10 weeks and weighing between 25 g and 30 g, were purchased from the Experimental Animal Center of Central South University (Changsha, China). All mice were maintained in specific pathogen-free housing. All animal experiments were approved by the Animal Care and Use Committee of Central South University. All experiments were conducted in accordance with the National Institutes of Health Guidelines for the Care and Use of Laboratory Animals.

### LPS/ALI Model and Experimental Design

The LPS-induced ALI model was implemented using methods described previously^28^,^29^. Briefly, after the administration of anaesthesia, the trachea was exposed. A catheter (Abbot, Wiesbaden, Germany) was inserted into the trachea though the mouth, and mice received an intratracheal injection of LPS (*Escherichia coli* 0111:B4; Sigma-Aldrich, St. Louis, MO, USA) at a dose of 4 mg/kg in 25 μl of PBS, followed by 200 μl of air to ensure deposition throughout each lung. Control mice received an intratracheal injection of 25 μl of PBS. After installation, the wounds were closed, and the mice were allowed to recover with free access to food and water. Twenty-four hours after PBS or LPS administration, the animals were sacrificed and the blood and lungs were collected. Subsequently, the trachea was exposed and the lungs were excised from the mice by opening the chest via a median sternotomy. The wet weight (W) of the left lung was measured using an electronic scale, followed by desiccation in an oven at 65 °C for 48 h to determine the dry weight (D). The water content was obtained by calculating the W/D weight ratio. The right lung was removed and fixed in 4% paraformaldehyde (PFA) for 24 h. The lungs were also lavaged with 0.5 ml of cold PBS-EDTA (0.5 M) each time through a 20-gauge catheter into the trachea, and a total of 1.5 ml of BALF was instilled and withdrawn from each mouse to detect total protein levels.

### DNase I Treatment

In a second set of animals, mice were randomized to a group receiving instillations consisting of 20 μl of 3 mg/ml DNase I (5 mg/kg; Roche) at time 0 and 10 h or vehicle control (8.77 mg/ml NaCl; 0.15 mg/ml CaCl2) twice through a 20-gauge angiocath (BD, Canada), followed by 200 μl of air each time.

### Haematoxylin and eosin staining

For histopathological analysis, the right lung lobes were fixed in 4%PFA and embedded in paraffin. Five-micron sections were placed onto glass slides and stained with haematoxylin and eosin (H&E).ALI severity was evaluated by assigning a semiquantitative histology score via a double-blind method as described previously^30^. Briefly, the histological index of lung damage included alveolar oedema, haemorrhage, alveolar septal thickening and leukocyte infiltration. Each item was divided into four grades ranging from 0 to 3 (0 = normal; 1 = mild; 2 = moderate; 3 = severe), and then a total ALI score was calculated.

### Quantifying BALF DNA and Protein Levels

BALF supernatant double-stranded DNA was quantified using Quant-iT PicoGreen^®^ (Invitrogen, Canada) following the manufacturer’s protocol. Protein concentrations in BALF were quantified by performing a bicinchoninic acid protein assay (Biomiga, USA).

### Neutrophil and Platelet Isolation

Mouse neutrophils were isolated from the bone marrow of the tibias and femurs using a commercially available mouse Neutrophil Isolation Kit (Miltenyi Biotec, Germany) according to the manufacturer’s instructions. The purity of the neutrophil preparations (consistently > 95%) was routinely verified with Giemsa staining, and cell viability (>97%) was verified by trypan blue exclusion assay.

Platelet isolation: anti-coagulated blood treated with citric acid-citrate-dextrose (ACD; 1:9 blood vol/vol) was obtained via cardiac puncture from isoflurane-anaesthetized mice. The sampling blood volume was approximately 1.2–1.5 ml per mouse. The samples were centrifuged at room temperature at 160 g for 10 min, and the platelet-rich plasma was collected and subsequently filtered through a sepharose 2B column equilibrated with 25 mM piperazine diethanesulfonic acid buffer (PIPES). The platelets were then centrifuged at room temperature for10 min at 650 g, harvested from the precipitate and resuspended in RPMI 1640.

### Identification of NETs *In Vivo* and *In Vitro*

In our *in vivo* experiments, paraffin-embedded mouse lungs were sectioned (4 μm), and sections were prepared and mounted on glass slides. After dewaxing, antigen retrieval was performed using citrate buffer and permeabilized with 0.1% Triton X-100 for 10 min, then specimens were blocked with PBS containing 3% bovine serum albumin (BSA) and 1% donkey serum. The sections were incubated with primary antibodies, specifically anti-citrullinated-histone H3 (1:100; Abcam) and anti-neutrophil elastase (1:50; Santa Cruz), followed by detection with Alexa Fluor 488 donkey anti-rabbit (1:500; Abcam) and Alexa Fluor 647 donkey anti-goat (1:500; Abcam) secondary antibodies overnight at 4 °C, respectively. 4′, 6-diamidino-2-phenylindole (DAPI) was applied to detect DNA.

In our *in vitro* experiments, neutrophils(10^5^) were plated and allowed to adhere to coated plates for 1 h prior to incubation with PMA(100 nM; Sigma-Aldrich), LPS(5 ug/ml), platelets(10^6^), or LPS with platelets for 4 h. Slides were incubated with the primary and secondary antibodies described above; the concentrations of primary antibody anti- Cit-H3, anti-neutrophil elastase, secondary antibodies Alexa Fluor 488 donkey anti-rabbit and Alexa Fluor 647 donkey anti-goat were 1:600, 1:100, 1:800 and 1:800,respectively. Slides were visualized with an Olympus Fluoroview 500 confocal microscope.

### Quantification of NETs

To quantify NET-DNA in cell culture supernatants and mouse plasma, a PicoGreen assay kit (Invitrogen) was employed.

### Immunoblotting

Western blot assays were performed using whole cell lysates obtained from lung tissue. Membranes were incubated overnight with the Cit-H3 (1:1000, Abcam) antibody and an anti-GAPDH antibody (1:2000, Proteintech) as an internal control.

### Quantification of Inflammatory Indicators

IL-6 and TNF-α levels in mouse plasma and BALF were determined using a commercially available mouse IL-6 and TNF-α enzyme-linked immunosorbent assay (ELISA) kit (RayBiotech, USA) according to the manufacturer’s instructions.

### Statistical Analysis

Results are expressed as the mean ± standard deviation(SD). Differences between more than two sets of data were assessed by performing one-way ANOVA followed by Tukey’s multiple-comparisons test. A value of P < 0.05 (two-tailed) was considered statistically significant.

## Additional Information

**How to cite this article**: Liu, S. *et al*. Neutrophil extracellular traps are indirectly triggered by lipopolysaccharide and contribute to acute lung injury. *Sci. Rep.* **6**, 37252; doi: 10.1038/srep37252 (2016).

**Publisher’s note:** Springer Nature remains neutral with regard to jurisdictional claims in published maps and institutional affiliations.

## Supplementary Material

Supplementary Information
