# Supplementary material for: Neutrophil extracellular traps are indirectly triggered by lipopolysaccharide and contribute to acute lung injury
Source: Sci Rep. 2016 Nov 16;6:37252. doi: 10.1038/srep37252 (PMC5110961; doi:10.1038/srep37252)
Supplement: Supplementary Information [file srep37252-s1.pdf]

Supplementary Information

Title: Neutrophil extracellular traps are indirectly triggered by lipopolysaccharide and contribute to acute lung injury

Manuscript#: SREP-16-31625A

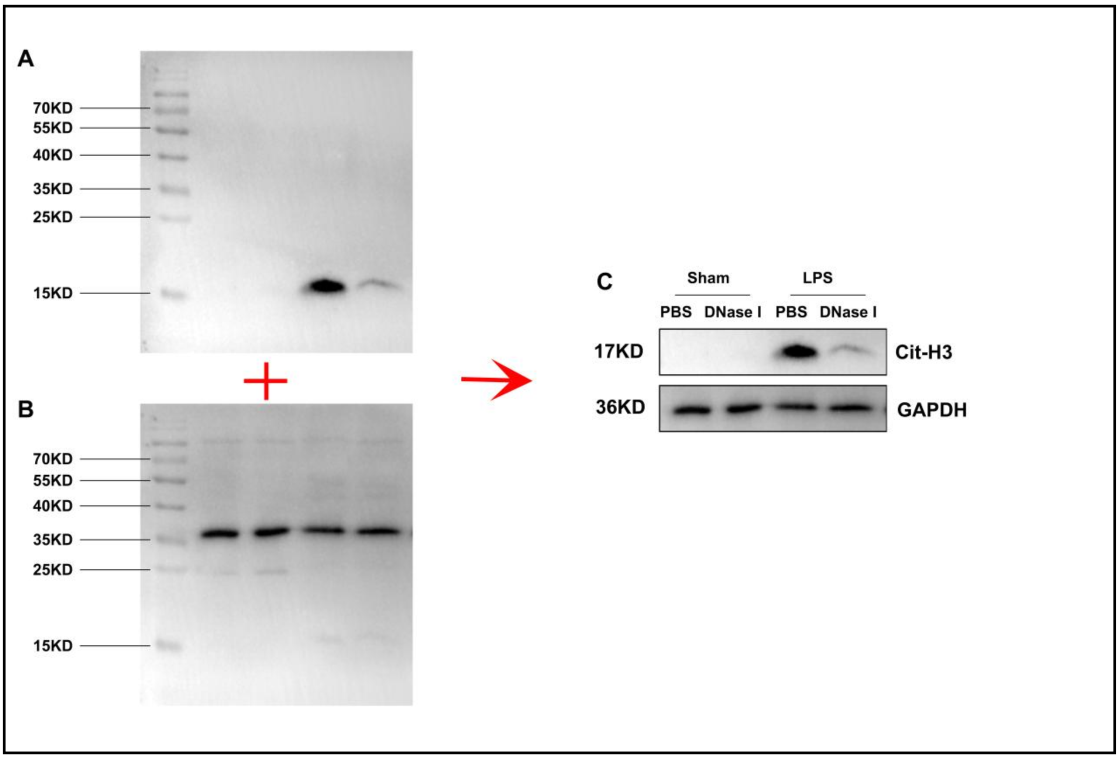

Figure S1: Western blot analysis of Cit-H3 protein levels in the lungs of sham-, LPS-, or DNase I plus LPS-treated mice. (A)Anti-Histone H3 (citrulline R2 + R8 + R17) antibody used at 1/1000 dilution detects a 17 kDa band in single lane Western Blot. (B)Anti-GAPDH antibody used at 1/2000 detects a 36 kDa band in Western Blot. (C) Cropped gels/blots are displayed.
